# *Mycobacterium tuberculosis* Rv0341 Promotes *Mycobacterium* Survival in In Vitro Hostile Environments and within Macrophages and Induces Cytokines Expression

**DOI:** 10.3390/pathogens9060454

**Published:** 2020-06-08

**Authors:** Abualgasim Elgaili Abdalla, Shuangquan Yan, Jie Zeng, Wanyan Deng, Longxiang Xie, Jianping Xie

**Affiliations:** 1 State Key Laboratory Breeding Base of Eco-Environment and Bio-Resource of the Three Gorges Area, Key Laboratory of Eco-Environments in Three Gorges Reservoir Region, Ministry of Education, School of Life Sciences, Institute of Modern Biopharmaceuticals, Southwest University, Beibei, Chongqing 400715, China; aealseddig@ju.edu.sa (A.E.A.); 13554239641@163.com (S.Y.); zengjie890110@126.com (J.Z.); dengwanyan2009@163.com (W.D.); 2 Department of Clinical Laboratory Sciences, Faculty of Applied Medical Sciences, Jouf University, Sakaka, Al Jouf 2014, Saudi Arabia; 3 Department of Preventive Medicine, Institute of Biomedical Informatics, Bioinformatics Center, Henan Provincial Engineering Center for Tumor Molecular Medicine, School of Basic Medical Sciences, Henan University, Kaifeng 475004, China; xielongxiang123@126.com

**Keywords:** *Mycobacterium tuberculosis*, Rv0341, stress, infection, macrophages, cytokines

## Abstract

*Mycobacterium tuberculosis* represents an ancient deadly human pathogen that can survive and multiply within macrophages. The effectors are key players for the successful pathogenesis of this bacterium. *M. tuberculosis* open reading frame (ORF) *Rv0341*, a pathogenic mycobacteria-specific gene, was found to be upregulated in macrophages isolated from human tuberculosis granuloma and inside the macrophages during in vitro infection by *M. tuberculosis*. To understand the exact role of this gene, we expressed the *Rv0341* gene in *M. smegmatis*, which is a non-pathogenic *Mycobacterium*. We found that Rv0341 expression can alter colony morphology, reduce the sliding capability, and decrease the cell wall permeability of *M. smegmatis*. Furthermore, Rv0341 remarkably enhanced *M. smegmatis* survival within macrophages and under multiple in vitro stress conditions when compared with the control strain. Ms_Rv0341 significantly induced expression of TNF-α, IL-1β, and IL-10 compared with *M. smegmatis* harboring an empty vector. In summary, these data suggest that Rv0341 is one of the *M. tuberculosis* virulence determinants that can promote bacilli survival in harsh conditions and inside macrophages.

## 1. Introduction

Tuberculosis (TB), caused by *Mycobacterium tuberculosis* (*Mtb*), which infects millions of people every year, remains one of the top ten leading causes of mortality worldwide [1].

*Mtb* is a tenacious human pathogen that can survive in hostile host microenvironments such as low pH, hypoxia, oxidative stress as well as several other antimicrobial factors. Moreover, *Mtb* can reside and multiply inside macrophages, which is considered as the front-line of innate defense against *Mtb* [2,3,4]. So far, several *Mtb* effectors have been observed to be strongly involved in bacilli survival within macrophages and hostile conditions [5,6], while many other *Mtb* open reading frames (ORFs) function or their contribution in *Mtb* pathogenesis remains largely unknown.

The *Mtb Rv0341* gene is predicted to encode glycine-rich cell wall protein similar to that of *Arabidopsis thaliana* (http://tuberculist.epfl.ch/), suggesting that the Rv0341 function is the cell wall stabilization [7]. The *Rv0341* gene is highly conserved among the *M. tuberculosis* complex, while its ortholog is absent in non-pathogenic *Mycobacterium species* [National Center for Biotechnology Information (NCBI) BLAST server]. *Rv0341* (iniB), along with *Rv0342* (iniA) and *Rv0343* (iniB), clustered in a single operon known as iniABC (Isoniazid-(INH)-Inducible gene A, B, and C), which was upregulated upon in vitro treatment with many cell wall biosynthesis targeting antimicrobial drugs [8,9], implicating that it may play a role in cell wall organization. Interestingly, unlike *iniA* and *iniC*, the *Rv0341* gene was found to be upregulated in macrophages isolated from both necrotic and nonnecrotic granuloma tissues obtained from TB patients [10], suggesting a potential role in *Mtb* adaptation or persistence in hostile microenvironments. Moreover, Rv0341 peptides can be detected within the phagosome of *Mtb*-infected macrophages and induce specific cytotoxic (CD8+) T cell response [11], indicating a role in host–pathogen interaction. However, the role of *Rv0341* in *Mtb* physiology or survival in hostile environments and macrophages remains elusive.

*M. smegmatis* is a fast-growing nonpathogenic *Mycobacterium* species that is considered to be an ideal surrogate model to understand *Mtb* physiology and the function of *Mtb* genes through generating recombinant strains [12,13,14]. Thus, we investigated the function of *Mtb Rv0341* by constructing a recombinant *M. smegmatis* expressing Rv0341 (Ms_Rv0341) and *M. smegmatis* (Ms_Vec) harboring empty vector as the control. We found that Rv0341 can promote *M. smegmatis* survival upon multiple in vitro stresses and inside the macrophages.

## 2. Materials and Methods

### 2.1. Bacterial Strains, Cells Line, and Culture Conditions

*Mycobacterium smegmatis* mc2155 strain was maintained in the Institute of Modern Biopharmaceuticals, Southwest University. *Mtb* H37Rv genomic DNA was obtained from Beijing Thoracic Hospital. The human leukemia monocytic cell line (THP-1) was bought from the Conservation Center in Wuhan University (China), and the murine RAW264.7 macrophage cell line was a kind gift from Zhang from the Institute of Immunology, Third Military University of PLA, Chongqing, China [15]. *M. smegmatis* mc2155 was sub-cultured in Middlebrook-7H9 (MB7H9) broth and MB7H10 agar containing 0.2% (w/v) glucose, 0.5% (v/v) glycerol, and 0.05% (v/v) Tween 80, and then incubated at 37 °C with or without shaking depending on the medium. Kanamycin (20 µg/mL) was also added as a selective agent for recombinant strains.

### 2.2. Cloning of Rv0341 and Construction of Recombinant Strains

*Mycobacterial* plasmid pNIT-1 containing Myc-tag was used in our study as previously described in [16]. *Mtb* ORF *Rv0341* gene 1440 bp was amplified by polymerase chain reaction (PCR) from *Mtb* H37Rv genomic DNA by using gene-specific primers as a followed forward primer (F): 5′-CGGCATATGATGAAGATGACCTCGC-3′ and backward primer (B): 5′-AATATGGATCCGAACCCGGGTAGTC-3′ (backward), nucleotides underlined are sites for restriction enzymes NdeI and BamH1, respectively. The PCR product was digested and subsequently cloned in pNIT-1 vector generating the pNIT-1–Rv0341. Next, pNIT-1–Rv0341 was transformed into the *M smegmatis* mc2155 by electroporation to produce *M. smegmatis* harboring the *Rv0341* gene (Ms_Rv0341). Similarly, we constructed the control strains by transferring empty pNIT-1 into the *M smegmatis* mc2155 (Ms_Vec). Ms_R0341 and Ms_Vec were grown on MB7H9 agar containing 20 μg/mL kanamycin.

### 2.3. Detection of the Expression of Rv0341

Ms_Rv0341 containing myc-tagged-*Rv0341* and Ms_Vec carrying the empty vector were inoculated into MB7H9 broth supplemented with 0.5% (v/v) glycerol and 0.05% (v/v) Tween-80, then the inoculum was incubated with shaking at 37 °C. When the growth of recombinant strains reached an OD_600_ ~0.8, the inducer epsilon-(ε)-caprolactam (Aladdin, Shanghai, China) was added with a final concentration of 28 mM, then, the cultures were reincubated for 24 h. Next, the bacterial cells were harvested by using cold centrifugation with a speed of 3000× g for 10 min. The collected bacteria were washed four times with sterile, cold phosphate buffer saline (PBS). Next, supernatants were discarded safely, and the sediments were suspended in sterile, cold PBS and lysed by ultrasonication. SDS-PAGE and the western blot technique were used to analyze the bacterial lysates. Myc-tagged-Rv0341 was demonstrated using mouse IgG specific to Myc-tag-protein (TIANGEN, Beijing, China), and observed following treatment with a horseradish peroxidase-labeled anti-mouse IgG monoclonal antibody (IgG-HRP) (TIANGEN, Beijing, China).

### 2.4. In Vitro Growth Kinetic Analysis

In vitro growth kinetics was performed by inoculating the recombinant *M. smegmatis* strains in triplicate onto MB7H9 broth with a starting optical density (OD600 ~0.03). The culture was then placed in a shaker incubator at 37 °C. The inducer ε-caprolactam was added with a final concentration of 28 mM to the bacteria at a growth density of an OD_600_ ~0.8. The growth curve was plotted by measuring bacterial density (OD_600_ values) at 6 h intervals throughout the 58 h.

### 2.5. Colony Morphology and Sliding Motility Analysis

For colony morphology analysis, log-phase growth of Ms_Rv0341 and Ms_Vec was plated onto MB7H9 agar supplemented with a ε-caprolactam inducer at a final concentration of 28 mM and the result was obtained within 5–6 days of incubation at 37 °C.

The bacterial sliding motility was analyzed via a method previously described by Huang et al. [17]. Briefly, the mid-exponential growth of Ms_Rv0341 and Ms_Vec was treated with a ε-caprolactam inducer at a final concentration of 28 mM for up to 16 h and then the growth was collected and diluted with sterile MB 7H9 broth for up to an OD_600_ of ~0.5. Next, 3 μL of diluted bacterial growth was inoculated onto the middle of MB7H9 plates containing 0.3% agarose (Promega). The results obtained after 4–5 days of plate incubation at 37 °C.

### 2.6. Assessments of Recombinant Strains Survival under In Vitro Harsh Environments

Ms_Rv0341 and Ms_Vec were cultivated onto MB 7H9 broth supplemented with 20 μg/mL kanamycin. When their growth reached an OD_600_ ~0.5, ε-caprolactam was added to a final concentration of 28 mM and reincubated at 37 °C with shaking for 16 h before being exposed to stress conditions.

The survival of recombinant *M. smegmatis* strains in low pH stress was measured as previous report [18]. In summary, the induced bacterial growth was collected, washed with acidic MB7H9 medium (pH 5 or 3), and then the bacteria were suspended in 10 mL of acidic MB7H9 broth (pH 5 or 3) to an OD_600_ of 0.5. After culture incubation at 37 °C with shaking, 100 μL was taken, serially diluted, and plated on MB7H9 agar to count the viability of bacteria at 0, 3, 6, and 9 h.

To evaluate bacilli survival in surface stress [19], Ms_Rv0341 and Ms_Vec growth was induced with ε-caprolactam, then collected and washed twice with sterile MB7H9 broth. The sediment was suspended in sterile MB7H9 broth for up to an OD_600_ of ~0.5 and treated with SDS to a final concentration of 0.05%. A 100 μL of the sample was extracted at 0, 2, 3, and 4 h post-incubation at 37 °C with shaking. The serially diluted samples were inoculated onto MB7H9 agar plates and incubated at 37 °C for 4–5 days to count the colony forming units (CFUs).

The tolerance of Ms_RV0341 and Ms_Vec strains to oxidative stress was examined using hydrogen peroxide (H_2_O_2_) and diamide. To compare the survival of bacteria under H_2_O_2_ stress [20], ε-caprolactam induced recombinant *M. smegmatis* strains growth was harvested, washed, and diluted with sterile MB7H9 broth for up to an OD_600_ of ~0.5. Next, 10 mL from each strain suspension was placed in a sterile tube and treated with H_2_O_2_ at a final concentration of 7 mM. Then, the H_2_O_2_ treated cultures were incubated at 37 °C with shaking. A 100 μL from each culture was taken at 0, 60, and 120 min and then, 10-fold serially diluted from 10^−1^ to 10^−5^ with sterile PBS and 10 μL of the suspension was plated onto MB7H9 agar. The result was obtained after four days of incubation at 37 °C. The survival of recombinant strains under diamide stress was examined, as previously conducted in [21]. In brief, after 16 h of induction of Ms_Vec and Ms_Rv0341 growth with 28 mM ε-caprolactam, the bacteria were gathered, washed, and suspended in sterile MB7H9 broth for up to an OD_600_ of ~0.8. After that, the bacteria was 10-fold serially diluted from 10^−1^ to 10^−5^ with sterile PBS, and 10 μL of bacteria suspension was spotted onto MB7H9 agar plates supplemented with 28 mM of ε-caprolactam and diamide at a final concentration of 2 mM or 7 mM. In addition, the recombinant strains were inoculated into MB7H9 agar plates free from diamide as the negative control. The measurement was performed after incubation of the inoculated plates at 37 °C for four days.

All stress experiments were done in three biological repeats and three independent experiments.

### 2.7. Cell Wall Permeability Analysis

*Mycobacterium* cell wall permeability was analyzed by measuring the accumulation rate of fluorescence dyes, namely, ethidium bromide (EtBr) and Nile Red [22,23]. In brief, the recombinant *M. smegmatis* strains were inoculated onto M7H9 broth and incubated at 37 °C in a shaker incubator. When the growth density of Ms_Rv0341 and Ms_Vec reached an OD_600_ of ~0.5, the ε-caprolactam was added at a final concentration of 28 mM and the culture reincubated for up to 16 h. After that, the recombinant *M. smegmatis* strains were harvested, washed using sterile PBS solution containing 0.05% Tween 80 (PBST), and then, resuspended in sterile PBS to adjust their density to an OD_600_ of ~0.5. The bacterial suspensions were dispensed in triplicate in microliter plates and treated with EtBr and Nile Red dye to a final concentration of 2 μg/mL and 20 μM, respectively. The fluorescence intensity of accumulated dyes was measured at limited time points by excitation at 544 nm and emission at 590 nm using a FLUOstar OPTIMA microplate reader (BMG Labtech, Ortenberg, Germany).

### 2.8. M. smegmatis Strains Infection of Macrophages

THP-1 (human monocytic cell line) was cultured at 1 × 10^6^ cells for each well in 6-well culture cell line plates. Differentiation of THP-1 cells into macrophages was stimulated by the addition of 20 nM of phorbol 12-myristate 13-acetate (PMA) (Sigma, St. Louis, MO, USA) for 48 h. Then, THP-1 macrophages infected with *M. smegmatis* expressing Rv0341 (Ms_Rv0341) and the control strain (Ms_Vec) at the multiplicity of infection (MOI) equal to 10:1 (bacteria-to-macrophage ratio) and incubated at 37 °C in 5% carbon dioxide. The extracellular bacteria were removed after four hours of infection by washing the cell lines three times with a warm RPMI-1640 medium (Hyclone, Logan, UT, USA). Then, warm RPMI-1640 supplemented with 10% fetal bovine serum (Hyclone), 250 nM IVN (Sigma) suspended in dimethyl sulfoxide, and 100 mg/mL hygromycin B antibiotic (Roche, Branchburg, NJ, USA) were added. For intracellular bacteria survival assay, THP-1 macrophages were washed twice with sterile PBS and lysed with 1 mL of 0.025% SDS (w/v). The cell lysate was serially diluted and inoculated onto MB7H9 agar supplemented with 20 μg/mL kanamycin. The colony-forming units (CFU) of bacteria was counted after four days of incubation at 37 °C.

The intracellular survival of Ms_Rv0341 and Ms_Vec in RAW264.7 murine macrophages was also performed by the same method above-mentioned for THP-1 macrophage.

### 2.9. Analysis of Cytokine Expression

The total RNA was extracted at 6 and 48 h from post-infected THP-1 macrophages with Ms_Rv0341 and Ms_Vec using RNA extraction kit (TIANGEN), according to the manufacturer’s directions. Next, RNA was converted into cDNA by reverse transcriptase-PCR by using the PrimeScript RT reagent kit (Takara, Shiga, Japan). The mRNA levels of tumor necrosis factor-alpha (TNF-α), interleukin-1beta (IL-1β), interleukin-6 (IL-6), interleukin-10 (IL-10), and beta-actin were measured by quantitative RT-PCR. The reactions were conducted in the CFX96 Touch RT-PCR Detection System (Bio-Rad) using the SYBR Green Master mix and target gene-specific primers (Table 1). The relative cytokine expression levels were determined after normalized to the level of beta-actin expression.

### 2.10. Data Analysis

Data analysis was performed using Graphpad Prism software version 6 (GraphPad, San Diego, CA, USA), and statistical significance between the studied groups was determined by the student’s t-test. The result showed that a P-value less than 0.05 was considered significant and represented by the asterisk as * *p* < 0.05, ** *p* < 0.01, and *** *p* < 0.001.

## 3. Results

### 3.1. M. tuberculosis Rv0341 Protein Ectopically Expressed in M. smegmatis

**Figure 1:**
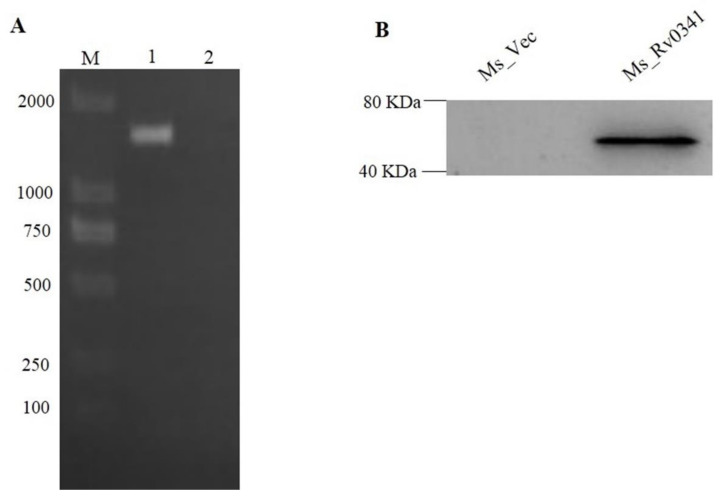
Rv0341 protein was ectopically expressed in *M. smegmatis*. (**A**) The 1440 bp *Rv0341* gene detected in Ms_Rv0341, Lane M (DNA ladder marker), Lane 1 is the band of *Rv0341* gene, and Lane 2 is the negative control (Ms_Vec). (**B**) Western blot detection of Rv0341 protein expression in *M. smegmatis*.

To understand the exact function of *Rv0341* in *Mycobacterium*, we first constructed recombinant *M. smegmatis* strains (Ms_Rv0341) by expressing the Myc-tagged Rv0341 protein from the pINT-1-vector. The control strain Ms_Vec was generated by the transformed empty pINT-1 vector only. To examine the successful cloning and expression of Rv0341, Ms_Rv0341 and Ms_Vec were cultured on MB7H9 medium containing 20 μg/mL kanamycin. The successful cloning of the *Rv0341* gene was affirmed by performing PCR from bacterial colonies (Figure 1A). The recombinant *M. smegmatis* strains were lysed by ultrasonication after 24 h of induction with ε-caprolactam. Rv0341-Myc-tagged protein expression was present as 43.9 kDa in total Ms_ Rv0341 strain lysate by western blot, but it was absent in the lysate of Ms_Vec (Figure 1B).

### 3.2. Rv0341 Alters the Colony Morphology and Sliding Motility of M. smegmatis

**Figure 2:**
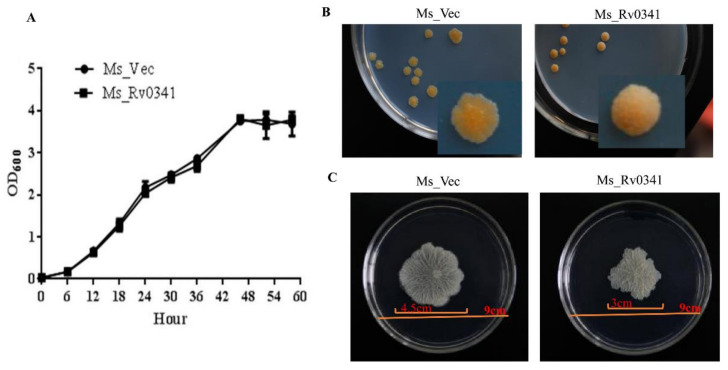
Ms_Rv0341 colonial morphology was altered and its sliding motility was reduced. (**A**) In vitro growth kinetics of Ms_Rv0341 and Ms_Vec strains in liquid MB7H9 medium was determined by measuring growth optical density (OD_600_) every 6 h. (**B**) The colony of Ms_ Rv0341 and Ms_Vec on MB7H9 agar. (**C**) Sliding of recombinant *M. smegmatis* strains in MB7H9 containing 0.03% agarose. The photographs were taken at 5–6 days after incubation at 37 °C.

To analyze the effects of Rv0341 on the growth of *Mycobacterium*, the growth phenotype of the recombinant *M. smegmatis* strains was monitored. Ms_Rv0341 and Ms_Vec have similar in vitro growth kinetics (Figure 2A), suggesting that Rv0341 did not affect the growth rate of *Mycobacterium*. However, the Ms_Rv0341 colonial morphology altered into being dome-like and was rougher compared with the flat, irregular margins and less rough colonies of the Ms_Vec (Figure 2B). In addition, Ms_Rv0341 sliding motility was remarkably reduced when compared with the control strain (Figure 2C). These data suggest that Rv0341 could modify the mycobacterial cell envelope properties.

### 3.3. Rv0341 Mediates Mycobacterium Resistance to Harsh Environments

**Figure 3:**
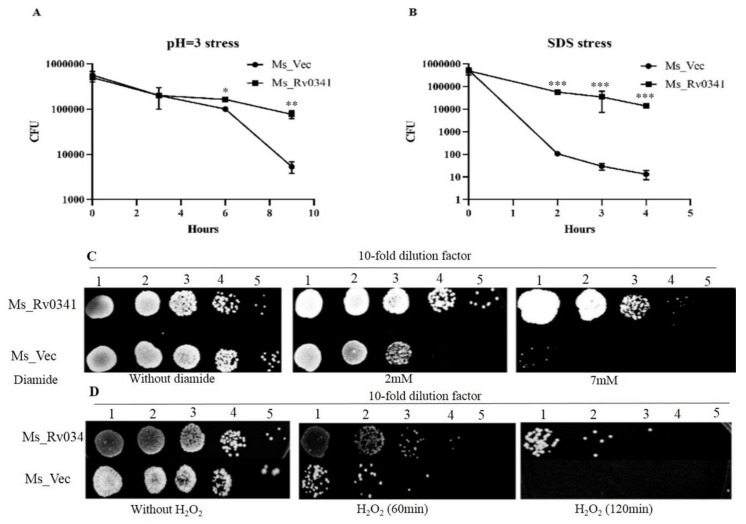
Rv0341 can enhance the Mycobacterium resistance to multiple stresses. (**A**) Survival of Ms_Vec and Ms_Rv0341 strain under pH 3. (**B**) Susceptibility of Ms_Vec and Ms_Rv0341 to the surface stressor (0.05% SDS). (**C**,**D**) Survival of Ms_Vec and Ms_Rv0341 in oxidative stress, (**C**) Survival of recombinant strains in the presence of diamide at a final concentration of 2mM or 7mM. (**D**) Survival of Ms_Rv0341 and Ms_Vec upon exposure to 7 mM hydrogen peroxide (H_2_O_2_). Asterisks represent statistical significant difference (* *p* < 0.05, ** *p* < 0.01, *** *p* < 0.001). Experiments were done in triplicate, and similar findings were obtained in three independent experiments.

*Rv0341* transcription was upregulated in macrophages isolated from the granuloma of TB patients, suggesting a role in the survival of the pathogen in harsh microenvironments [10]. In addition, alteration of *M. smegmatis* colony morphology was associated with resistance to stress [14]. Therefore, we analyzed the effect of Rv0341 on *Mycobacterium* survival in multiple in vitro stresses, where Ms_Rv0341 and Ms_Vec showed similar survival rate under a pH 5 stress condition (data not shown). Nevertheless, Ms_Rv0341 was significantly more resistance to pH 3 stress than the Ms_Vec strain at 6 and 9 h upon acid treatment (Figure 3A). Ms_Rv0341 survival was higher than the Ms_Vec (Figure 3B) upon exposure to 0.05% SDS. This was true for the exposure to diamide (Figure 3C) and H_2_O_2_ (Figure 3D). These data suggest that Rv0341 can enhance the *Mycobacterium* resistance to various stresses.

### 3.4. Rv0341 Decreases the Cell Wall Permeability of Mycobacterium

**Figure 4:**
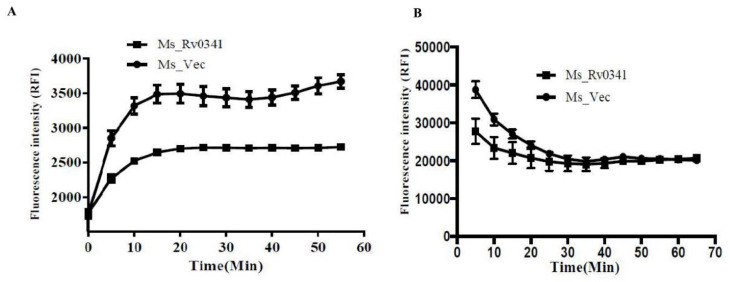
Rv0341 reduced the cell wall permeability of *Mycobacterium*. Accumulation of ethidium bromide (EtBr) and Nile Red dyes were used to analyze the cell envelope permeability of recombinant *M. smegmatis* strains. The ε-caprolactam-induced Ms_Rv0341 and Ms_Vec growth were collected, washed three times, and suspended in sterile phosphate buffer saline (PBS) to an OD600 of ~0.8. Next, the bacterial suspension was treated with 2 μg/mL EtBr (**A**,**B**) 20 μM Nile Red dye, and the fluorescence intensity was measured in appointed time using a FLUOstar OPTIMA microplate reader.

The ability of *Mtb* to survive in hostile environments was largely attributed to the impenetrable nature of its cell wall to toxic substances [24]. To evaluate whether Rv0341 expression affected the cell wall permeability of *M. smegmatis*, we measured the accumulation rate of fluorescence dyes, EtBr, and Nile Red in the Ms_Rv0341 and Ms_Vec strains. We found that the accumulation of EtBr dye was pronouncedly lower in the Ms_Rv0341 strain compared with the Ms_Vec strain (Figure 4A), indicating that Rv0341 expression reduced the cell wall permeability of *M. smegmatis*. While a lipid stain, Nile Red dye exhibited rapid and higher accumulation during the first 15 min in the Ms_Vec when compared with the Ms_Rv0341 strain (Figure 4B), suggesting that Rv0341 may delay the accessibility of Nile Red dye to the cell wall lipids. Thus, Rv0341 can alter the cell wall permeability.

### 3.5. Rv0341 Expression Increases Mycobacterium Survival in Macrophages

**Figure 5:**
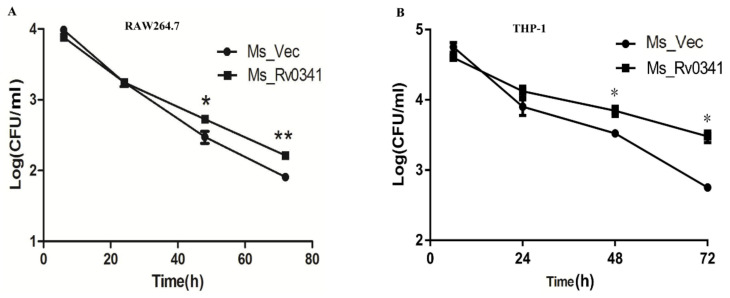
Rv0341 expression increased Mycobacterial survival within macrophages. (**A**) RAW267.4 murine macrophages and (**B**) phorbol 12-myristate 13-acetate (PAM)-differentiated THP-1 macrophages were infected with Ms_Vec and Ms_Rv0431. The infected macrophages were washed by sterile PBS and then lysed with 1 mL of 0.025% SDS at the indicated time. Next, cell lysate was serially diluted and inoculated on kanamycin MB7H9 agar to enumerate the bacterial colony-forming units (CFU/mL). Asterisks indicated statistical significance; * *p* < 0.05; ** *p* < 0.01. The experiments were done in three biological repeats, and similar findings were achieved in three independent experiments.

*Rv0341* has been found expressed within macrophages isolated from the granuloma of TB patients [10], and the Rv0341 protein can be detected upon in vitro infection of the macrophages by *Mtb* [11]. To test whether Rv0341 can affect *Mycobacterium* survival within macrophages, the intracellular survivability of Ms_Rv0341 and Ms_Vec was monitored in RAW264.7 macrophages and THP-1 macrophages. Four hours post-infection, the extracellular bacteria were eliminated via washing three times with RPMI-1640 medium and treating with antibiotic hygromycin B. Ms_Rv0341 survival was higher in both murine RAW264.7 macrophages (Figure 5A) and THP-1 macrophages (Figure 5B) at 48 and 72 h of post-infection than those of the Ms_Vec strain. These data suggest that Rv0341 is a virulence factor that can promote *Mycobacterium* intracellular survival within macrophages.

### 3.6. Rv0341 Induced Innate Cytokines Expression

**Figure 6:**
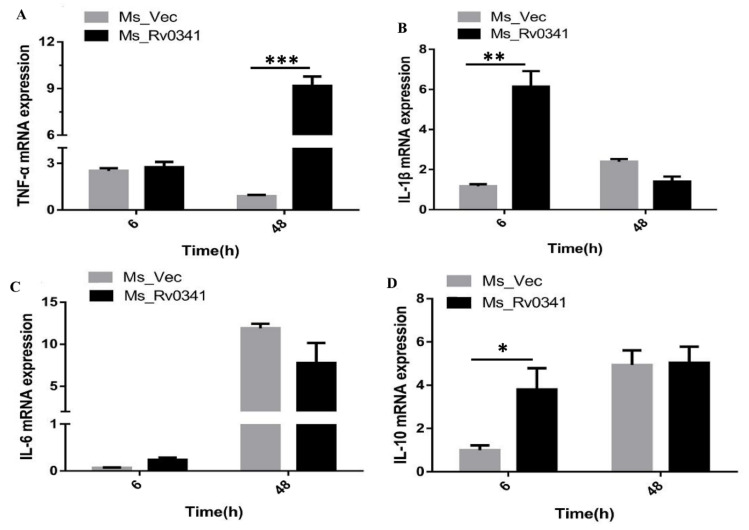
Ms_Rv0341induced cytokine expression. The cytokine transcription of THP-1 macrophages infected with Ms_Rv0341 or Ms_Vec was examined. Total RNA was extracted at 6 and 48 h of post-infection, and subsequently, cDNA was generated and analyzed by quantitative qRT-PCR. (**A**) TNF-α, (**B**) IL-1β, (**C**) IL-6, and (**D**) IL-10 expressions were calculated after being normalized with expression levels of beta-actin. Asterisks indicate significant difference as * *p* < 0.05, ** *p* < 0.01, *** *p* < 0.001. The experiments were done in triplicates, and similar findings were obtained in three independent experiments.

Rv0341 can induce specific cytotoxic (CD8+) T cell response, and cytokines released [11,25]. To explore whether Rv0341 can promote the expression of innate immunity cytokines, the expression of TNF-α, IL-1β, IL-6, and IL-10 were analyzed during infection of THP-1 macrophages with Ms_Vec and Ms_Rv0341. Total RNA was exacted from infected macrophages at 6, and 48 h and mRNA levels were measured by qRT-PCR. The results demonstrate that infection with Ms_Rv0341 significantly induces the transcription of TNF-α, IL-1β, and IL-10 in THP-1 macrophages when compared to the Ms_Vec strain, as shown in Figure 6A,B,D, respectively. However, there was no significant difference for the IL-6 transcription between Ms_Rv0341 and Ms_Vec (Figure 6C). These findings indicate that Rv0341 can promote innate cytokine expression.

## 4. Discussion

*Mtb* can hijack host immune defenses to persist within the host for years, a big challenge for TB eradication [26,27]. Understanding the *Mtb* effectors involved in this interaction can inform better control measures against tuberculosis.

Previous studies reported that drugs targeting the cell wall biosynthesis can induced expression of *Rv0341* (iniB), *Rv0342* (iniA), and *Rv0343* (iniB) as single operon called the iniABC operon [8,9], suggesting a potential role in cell wall protection. Furthermore, iniABC operon transcription was reported to be upregulated by the cell envelope stress regulator Rv0339c protein [28], but *Rv0341* was the only gene of the iniABC operon regulated by the stress response regulator MT2816/Rv2745c [29], another example of its involvement in the stress response. *Rv0341* was also the only member of the iniABC operon that was expressed in macrophages harvested from the central zone of granuloma tissue from TB patients, suggesting a role in *Mtb* survival in hostile microenvironments [10]. Nevertheless, the role of Rv0341 in *Mtb* remains elusive. By using the NCBI BLASTn server, we found that the *Rv0341* gene is highly conserved among the *M. tuberculosis* complex. Furthermore, *Rv0341* has no ortholog in *M. smegmatis*, *M. leprae*, *M. avium*, and other acid-fast bacilli such as *Streptomyces avermitilis* (Figure S1A). Rv0341 protein is predicted to contain an unknown function domain (Figure S1B). Therefore, we expressed Rv0341 in the *M. smegmatis* to study its function. We found that Rv0341 expression altered colony appearance and reduced the sliding motility of *M. smegmatis*. The alteration of *Mycobacterium* colony morphology was associated with enhanced survival in stresses and within macrophages [14,17,30]. Ms_Rv034 survival was increased upon multiple in vitro stresses including acidic conditions, surface stress, and oxidative stress. Ms_Rv0341 cell wall permeability was reduced, consistent with previous reports on the role of cell wall permeability in survival [31,32]. Rv0341 was present in the cell wall of *M. smegmatis* (Figure S2), consistent with the reported presence of Rv0341 in the *Mtb* H37Rv cell wall [33]. It has been proposed that Rv0341 may play a role in cell wall stabilization [7].

Interestingly, *Mtb Rv0341* was shown to be upregulated in macrophages isolated from the granuloma of TB patients [10] and Rv0341 was isolated from *Mtb* infected macrophages in an in vitro experiment [11], suggesting a role as a virulence factor. In addition, *Rv0341* transcription was higher in *Mtb* H37Rv than the SigD regulon mutant strain. This mutant strain exhibited less virulence in the mice model compared with wild type strain, indicating that SigD regulons are crucial for *Mtb* pathogenesis [34]. Nevertheless, its role in mycobacterial virulence or survival inside macrophages remains unknown. Our study showed that Ms_Rv0341 had a significantly higher bacillary load inside macrophages compared with the Ms_Vec, suggesting a role in withstanding intracellular stresses [35,36,37].

Rv0341 can induce specific cytotoxic T cell responses, which can recognize and destroy the *Mtb*-infected dendritic cells [11]. Rv0341 peptides enhanced T cell proliferation and the expressions of cytokines such as interferon-gamma (IFN-γ) and IL-2 [25]. It was also detected to react with the serum samples obtained from TB patients [38]. However, Rv0341 effects on innate immune cytokines remain elusive. We demonstrated that Rv0341 significantly induced early expression of IL-1β at 6 h of post-infection of the THP-1 macrophage. IL-1β is a proinflammatory cytokine that plays a dual role during *Mtb* infection. IL-1β is required for the early containment of infection via priming of T cell activation. However, prolonged IL-1β expression could lead to serious tissue injury [39,40]. Virulent *Mtb* strain can induce a significant amount of IL-1β compared with less virulent strains [41]. *Mtb* early secretory antigenic target-6KDa (ESAT-6) can induce IL-1β from the peripheral blood macrophages obtained from latent TB patients compared with the cells from active TB patients [42]. IL-10 is an anti-inflammatory cytokine that can prevent tissue damage even at low level [43]. IL-10 knockout mice exhibited lower bacillary load in the lungs than the wild type mice [44], suggesting a role of IL-10 in *Mtb* pathogenesis. Our study found that Ms_Rv0341 infection significantly induced the expression of IL-10 compared with the control strain. Previously, it was reported that IL-10 could promote *Mtb* survival and growth within macrophages [45]. TNF-α is a proinflammatory mediator that plays a primary role in granuloma formation and enhanced *Mtb* persistence within the host tissues [46]. TNF-α is also critical to maintain the *Mtb* dormancy, as blockage of TNF-α leads to the reactivation of latent TB [47,48,49]. In the present study, we found that Ms_Rv0341 stimulated a more significant expression of TNF-α than Ms_Vec. Elevated levels of TNF-α were linked with hyper-virulent *Mtb* strains and increased number of bacilli in the patient’s sputum [41].

In summary, we have demonstrated that the expression of Rv0341 in *M. smegmatis* leads to altered colony morphology, reduced sliding motility, and decreased cell wall permeability. Rv0341 promoted *M. smegmatis* survival in multiple in vitro stress conditions and inside macrophages. However, the experiments using *Rv0341* deleted *Mtb* strain can provide more accurate information about the function of this gene. Further studies are needed to understand the effect of Rv0341 on the viability of macrophages, and how it interplays with complex host signaling.

## Figures and Tables

**Table 1:** Primers used for detection of cytokine expression.

Target Gene	Primers	Sequence (5′→3′)
IL-1β	F	TTCAGGCAGGCCGCGTCAGTTGT
	R	TGTGAGTCCCGGAGCGTGCAGTT
IL-6	F	GCCTTCGGTCCAGTTGCCTTCT
	R	TGCCAGTGCCTCTTTGCTGCTTT
IL-10	F	ACCTGGGTTGCCAAGCCTTGT
	R	GCTCCACGGCCTTGCTCTTGTTT
TNFα	F	GGCGGTGCTTGTTCCT
	R	GCTACAGGCTTGTCACTCG
β-actin	F	GTGACGTTGACATCCGTAAAGA
	R	GTGACGTTGACATCCGTAAAGA

F, Forward; R, Reverse.

## Supplementary Materials

The following are available online at https://www.mdpi.com/2076-0817/9/6/454/s1, Figure S1: The protein domain of Rv0341 (A) and the structure of the surrounding genes of Rv0341 (B). PHA03379 means “EBNA-3A; Provisional”. Figure S2: Rv0341 expressed in the cell wall of *M. smegmatis*.

## References

[1] World Health Organization (2019). Global Tuberculosis Report 2019.

[2] Bhat K.H., Mukhopadhyay S. (2015). Macrophage takeover and the host–bacilli interplay during tuberculosis. Future Microbiol..

[3] Hmama Z., Peña-Díaz S., Joseph S., Av-Gay Y. (2015). Immunoevasion and immunosuppression of the macrophage by mycobacterium tuberculosis. Immunol. Rev..

[4] Dorhoi A., Kaufmann S.H. (2014). Perspectives on host adaptation in response to mycobacterium tuberculosis: Modulation of inflammation. Semin. Immunol..

[5] Smith I. (2003). Mycobacterium tuberculosis pathogenesis and molecular determinants of virulence. Clin. Microbiol. Rev..

[6] Madacki J., Fiol G.M., Brosch R. (2019). Update on the virulence factors of the obligate pathogen mycobacterium tuberculosis and related tuberculosis-causing mycobacteria. Infect. Genet. Evol..

[7] Wang M., Guo X., Yang X., Zhang B., Ren J., Liu A., Ran Y., Yan B., Chen F., Guddat L.W. (2019). Mycobacterial dynamin-like protein inia mediates membrane fission. Nat. Commun..

[8] Alland D., Steyn A.J., Weisbrod T., Aldrich K., Jacobs W.R. (2000). Characterization of the mycobacterium tuberculosis inibac promoter, a promoter that responds to cell wall biosynthesis inhibition. J. Bacteriol..

[9] Bogatcheva E., Hanrahan C., Chen P., Gearhart J., Sacksteder K., Einck L., Nacy C., Protopopova M. (2010). Discovery of dipiperidines as new antitubercular agents. Bioorg. Med. Chem. Lett..

[10] Fenhalls G., Stevens L., Moses L., Bezuidenhout J., Betts J.C., van Helden P., Lukey P.T., Duncan K. (2002). In situ detection of mycobacterium tuberculosis transcripts in human lung granulomas reveals differential gene expression in necrotic lesions. Infect. Immun..

[11] Flyer D.C., Ramakrishna V., Miller C., Myers H., McDaniel M., Root K., Flournoy C., Engelhard V.H., Canaday D.H., Marto J.A. (2002). Identification by mass spectrometry of cd8+-t-cell mycobacterium tuberculosis epitopes within the rv0341 gene product. Infect. Immun..

[12] Agrawal P., Miryala S., Varshney U. (2015). Use of mycobacterium smegmatis deficient in adp-ribosyltransferase as surrogate for mycobacterium tuberculosis in drug testing and mutation analysis. PLoS ONE.

[13] Altaf M., Miller C.H., Bellows D.S., O’Toole R. (2010). Evaluation of the mycobacterium smegmatis and bcg models for the discovery of mycobacterium tuberculosis inhibitors. Tuberculosis.

[14] Singh P., Rao R.N., Reddy J.R.C., Prasad R., Kotturu S.K., Ghosh S., Mukhopadhyay S. (2016). Pe11, a pe/ppe family protein of mycobacterium tuberculosis is involved in cell wall remodeling and virulence. Sci. Rep..

[15] Zhang Z., Zhang Z.-Y., Schittenhelm J., Wu Y., Meyermann R., Schluesener H.J. (2011). Parenchymal accumulation of cd163+ macrophages/microglia in multiple sclerosis brains. J. Neuroimmunol..

[16] Li W., Zhao Q., Deng W., Chen T., Liu M., Xie J. (2014). Mycobacterium tuberculosis rv3402c enhances mycobacterial survival within macrophages and modulates the host pro-inflammatory cytokines production via nf-kappa b/erk/p38 signaling. PLoS ONE.

[17] Huang Q., Luo H., Liu M., Zeng J., Abdalla A.E., Duan X., Li Q., Xie J. (2016). The effect of mycobacterium tuberculosis crispr-associated cas2 (rv2816c) on stress response genes expression, morphology and macrophage survival of mycobacterium smegmatis. Infect. Genet. Evol..

[18] Luo H., Zeng J., Huang Q., Liu M., Abdalla A.E., Xie L., Wang H., Xie J. (2016). Mycobacterium tuberculosis rv1265 promotes mycobacterial intracellular survival and alters cytokine profile of the infected macrophage. J. Biomol. Struct. Dyn..

[19] Deng W. (2015). Pe11 (rv1169c) selectively alters fatty acid components of mycobacterium smegmatis and host cell interleukin-6 level accompanied with cell death. Front. Microbiol..

[20] Li X., Wu J., Han J., Hu Y., Mi K. (2015). Distinct responses of mycobacterium smegmatis to exposure to low and high levels of hydrogen peroxide. PLoS ONE.

[21] Viswanathan G., Joshi S.V., Sridhar A., Dutta S., Raghunand T.R. (2015). Identifying novel mycobacterial stress associated genes using a random mutagenesis screen in mycobacterium smegmatis. Gene.

[22] Rodrigues L., Ramos J., Couto I., Amaral L., Viveiros M. (2011). Ethidium bromide transport across mycobacterium smegmatis cell-wall: Correlation with antibiotic resistance. BMC Microbiol..

[23] Chuang Y.-M., Bandyopadhyay N., Rifat D., Rubin H., Bader J.S., Karakousis P.C. (2015). Deficiency of the novel exopolyphosphatase rv1026/ppx2 leads to metabolic downshift and altered cell wall permeability in mycobacterium tuberculosis. MBio.

[24] Hett E.C., Rubin E.J. (2008). Bacterial growth and cell division: A mycobacterial perspective. Microbiol. Mol. Biol. Rev..

[25] Horváti K., Pályi B., Henczkó J., Balka G., Szabó E., Farkas V., Biri-Kovács B., Szeder B., Fodor K. (2019). A convenient synthetic method to improve immunogenicity of mycobacterium tuberculosis related t-cell epitope peptides. Vaccines.

[26] Bussi C., Gutierrez M.G. (2019). Mycobacterium tuberculosis infection of host cells in space and time. FEMS Microbiol. Rev..

[27] Zu Bentrup K.H., Russell D.G. (2001). Mycobacterial persistence: Adaptation to a changing environment. Trends Microbiol..

[28] Boot M., van Winden V.J., Sparrius M., van de Weerd R., Speer A., Ummels R., Rustad T., Sherman D.R., Bitter W. (2017). Cell envelope stress in mycobacteria is regulated by the novel signal transduction atpase inir in response to trehalose. PLoS Genet..

[29] Mehra S., Dutta N.K., Mollenkopf H.-J., Kaushal D. (2010). Mt2816 encodes a key mycobacterium tuberculosis stress-response regulator. J. Infect. Dis..

[30] Maan P., Kumar A., Kaur J., Kaur J. (2018). Rv1288, a two domain, cell wall anchored, nutrient stress inducible carboxyl-esterase of mycobacterium tuberculosis, modulates cell wall lipid. Front. Cell. Infect. Microbiol..

[31] Campodónico V.L., Rifat D., Chuang Y.-M., Ioerger T.R., Karakousis P.C. (2018). Altered mycobacterium tuberculosis cell wall metabolism and physiology associated with rpob mutation h526d. Front. Microbiol..

[32] Kumari B., Saini V., Kaur J., Kaur J. (2020). Rv2037c, a stress induced conserved hypothetical protein of mycobacterium tuberculosis, is a phospholipase: Role in cell wall modulation and intracellular survival. Int. J. Biol. Macromol..

[33] Wolfe L.M., Mahaffey S.B., Kruh N.A., Dobos K.M. (2010). Proteomic definition of the cell wall of mycobacterium tuberculosis. J. Proteome Res..

[34] Raman S., Hazra R., Dascher C.C., Husson R.N. (2004). Transcription regulation by the mycobacterium tuberculosis alternative sigma factor sigd and its role in virulence. J. Bacteriol..

[35] Meena L.S. (2010). Survival mechanisms of pathogenic mycobacterium tuberculosis h37rv. FEBS J..

[36] Purdy G.E., Niederweis M., Russell D.G. (2009). Decreased outer membrane permeability protects mycobacteria from killing by ubiquitin-derived peptides. Mol. Microbiol..

[37] Deng W., Long Q., Zeng J., Li P., Yang W., Chen X., Xie J. (2017). Mycobacterium tuberculosis pe_pgrs41 enhances the intracellular survival of m. Smegmatis within macrophages via blocking innate immunity and inhibition of host defense. Sci. Rep..

[38] Li Y., Zeng J., Shi J., Wang M., Rao M., Xue C., Du Y., He Z.-G. (2010). A proteome-scale identification of novel antigenic proteins in mycobacterium tuberculosis toward diagnostic and vaccine development. J. Proteome Res..

[39] Marinho F.V., Fahel J.S., Scanga C.A., Gomes M.T.R., Guimarães G., Carvalho G.R., Morales S.V., Báfica A., Oliveira S.C. (2016). Lack of il-1 receptor–associated kinase-4 leads to defective th1 cell responses and renders mice susceptible to mycobacterial infection. J. Immunol..

[40] Zhang G., Zhou B., Li S., Yue J., Yang H., Wen Y., Zhan S., Wang W., Liao M., Zhang M. (2014). Allele-specific induction of il-1β expression by c/ebpβ and pu. 1 contributes to increased tuberculosis susceptibility. PLoS Pathog..

[41] Tram T.T., Nhung H.N., Vijay S., Hai H.T., Thu D.D., Ha V.T., Dinh T.D., Ashton P.M., Hanh N.T., Phu N.H. (2018). Virulence of mycobacterium tuberculosis clinical isolates is associated with sputum pre-treatment bacterial load, lineage, survival in macrophages, and cytokine response. Front. Cell. Infect. Microbiol..

[42] Lee M.-R., Chang L.-Y., Chang C.-H., Yan B.-S., Wang J.-Y., Lin W.-H. (2019). Differed il-1 beta response between active tb and ltbi cases by ex vivo stimulation of human monocyte-derived macrophage with tb-specific antigen. Dis. Markers.

[43] Abdalla A.E., Lambert N., Duan X., Xie J. (2016). Interleukin-10 family and tuberculosis: An old story renewed. Int. J. Biol. Sci..

[44] Redford P.S., Boonstra A., Read S., Pitt J., Graham C., Stavropoulos E., Bancroft G.J., O’Garra A. (2010). Enhanced protection to mycobacterium tuberculosis infection in il-10-deficient mice is accompanied by early and enhanced th1 responses in the lung. Eur. J. Immunol..

[45] O’Leary S., O’Sullivan M.P., Keane J. (2011). Il-10 blocks phagosome maturation in mycobacterium tuberculosis–infected human macrophages. Am. J. Respir. Cell Mol. Biol..

[46] Lin P.L., Plessner H.L., Voitenok N.N., Flynn J.L. (2007). Tumor Necrosis Factor and Tuberculosis. J. Investig. Dermatol. Symp. Proc..

[47] Kumar A., Lewin A., Rani P.S., Qureshi I.A., Devi S., Majid M., Kamal E., Marek S., Hasnain S.E., Ahmed N. (2013). Dormancy associated translation inhibitor (datin/rv0079) of mycobacterium tuberculosis interacts with tlr2 and induces proinflammatory cytokine expression. Cytokine.

[48] Dutta N.K., Illei P.B., Jain S.K., Karakousis P.C. (2014). Characterization of a novel necrotic granuloma model of latent tuberculosis infection and reactivation in mice. Am. J. Pathol..

[49] Dambuza I., Keeton R., Allie N., Hsu N.-J., Randall P., Sebesho B., Fick L., Quesniaux V.J., Jacobs M. (2011). Reactivation of m. Tuberculosis infection in trans-membrane tumour necrosis factor mice. PLoS ONE.

