# Supplementary material for: Mycobacterium tuberculosis Rv0341 Promotes Mycobacterium Survival in In Vitro Hostile Environments and within Macrophages and Induces Cytokines Expression
Source: Pathogens. 2020 Jun 8;9(6):454. doi: 10.3390/pathogens9060454 (PMC7350357; doi:10.3390/pathogens9060454)
Supplement: Supplementary file 1 [file pathogens-09-00454-s001.ppt]

## Slide 1
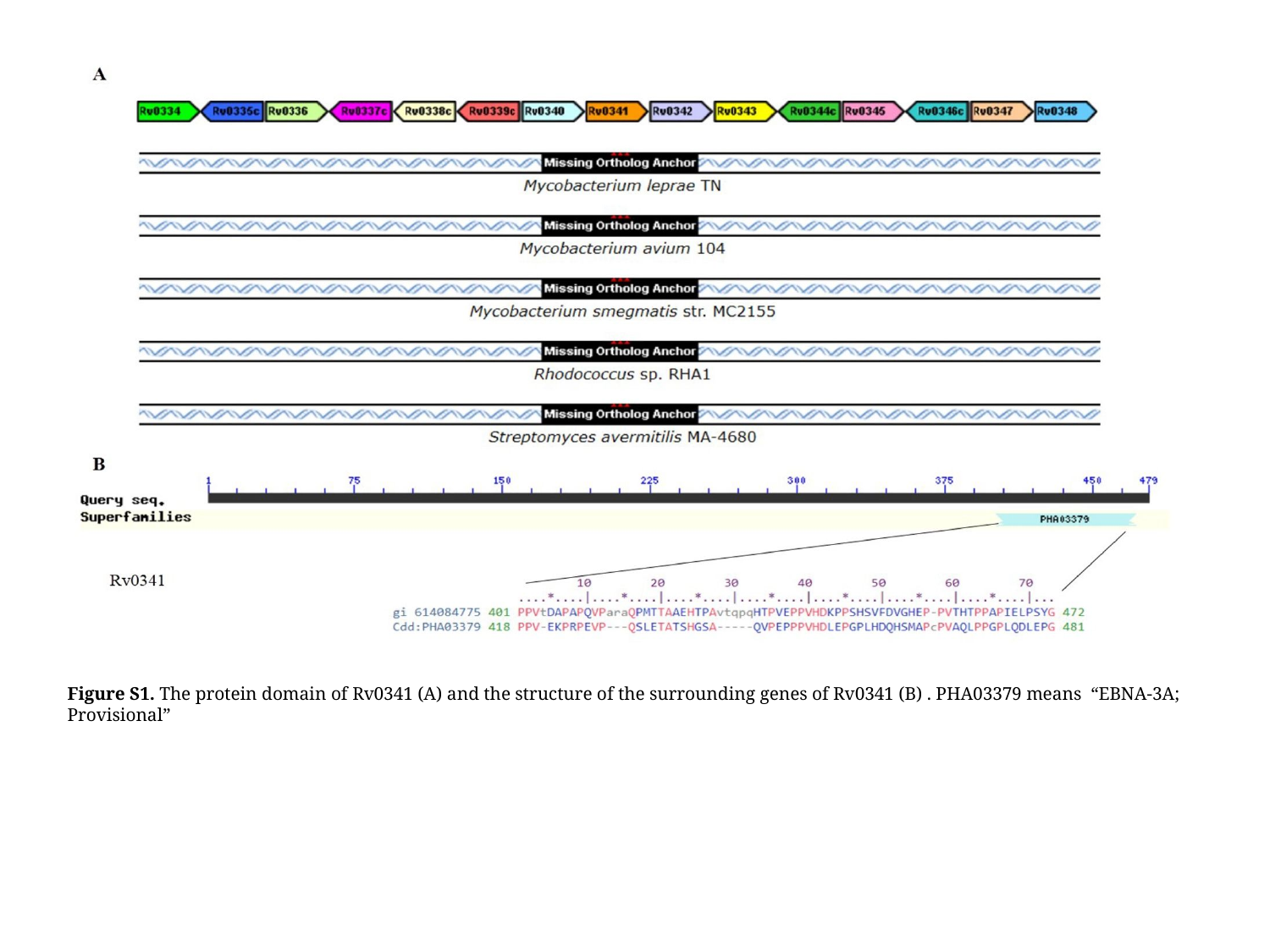

Figure S1. The protein domain of Rv0341 (A) and the structure of the surrounding genes of Rv0341 (B) . PHA03379 means “EBNA-3A; Provisional”

## Slide 2
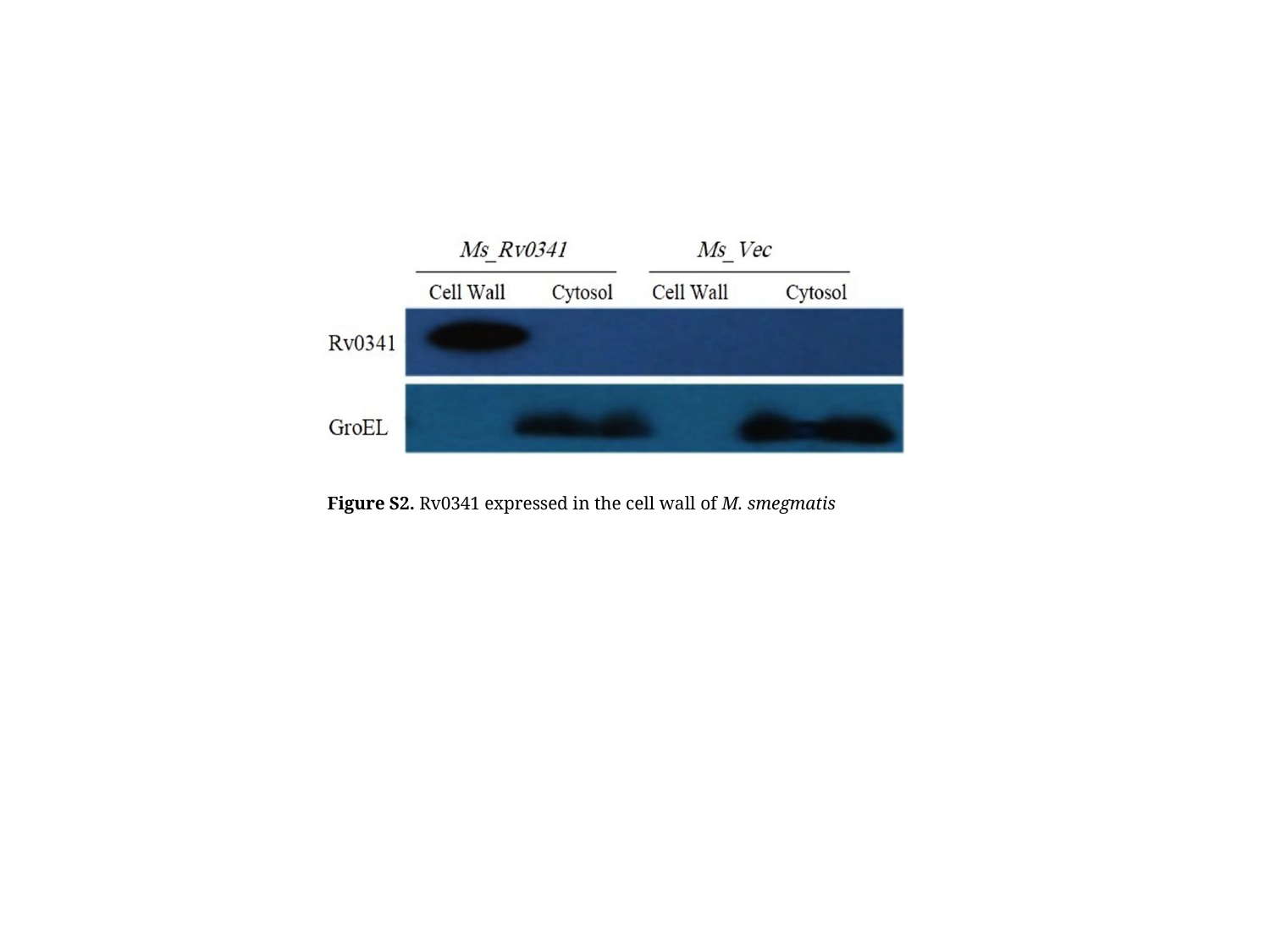

Figure S2. Rv0341 expressed in the cell wall of M. smegmatis
